# Structural Dynamics of Chloromethanes through Computational Spectroscopy: Combining INS and DFT

**DOI:** 10.3390/molecules27217661

**Published:** 2022-11-07

**Authors:** Mariela M. Nolasco, Mariana Matos Coimbra, Stewart F. Parker, Pedro D. Vaz, Paulo J. A. Ribeiro-Claro

**Affiliations:** 1 CICECO—Instituto de Materiais de Aveiro, Departamento de Química, Universidade de Aveiro, 3810-193 Aveiro, Portugal; 2 ISIS Neutron & Muon Source, STFC Rutherford Appleton Laboratory, Chilton, Didcot, Oxfordshire OX11 0QX, UK; 3 Champalimaud Foundation, Champalimaud Centre for the Unknown, 1400-038 Lisboa, Portugal

**Keywords:** chloroform, carbon tetrachloride, periodic-DFT, lattice modes, Fermi resonance

## Abstract

In this work, the structural dynamics of the chloromethanes CCl_4_, CHCl_3_ and CH_2_Cl_2_ were evaluated through a computational spectroscopy approach by comparing experimental inelastic neutron scattering (INS) spectra with the corresponding simulated spectra obtained from periodic DFT calculations. The overall excellent agreement between experimental and calculated spectra allows a confident assignment of the vibrational features, including not only the molecular fundamental modes but also lattice and combination modes. In particular, an impressive overtone sequence for CHCl_3_ is fully described by the simulated INS spectrum. In the CCl_4_ spectrum, the splitting of the ν3 mode at ca. 765–790 cm^−1^ is discussed on the basis of the Fermi resonance vs. crystal splitting controversy.

## 1. Introduction

Computational spectroscopy has become an invaluable tool to interpret spectroscopic results (see, e.g., previous work by the authors [1,2,3,4,5,6,7,8] and a recent overview on “computational molecular spectroscopy” [9]). Historically, the description of vibrational spectra often received some support from calculations, but in the last two decades or so has evolved from the mostly empirical approach, based on the comparison of similar molecules, to the current full “computational spectroscopy” era. Regarding vibrational spectra of molecular crystals, periodic-DFT calculations set the ground for detailed analysis, reliable assignments and deep interpretation.

Infrared and Raman spectra of chloromethane derivatives have long been subjects of detailed assignments, in particular, the molecular vibrations for samples in the gas and liquid phase [10]. Reports on the vibrational spectra of the crystals are also abundant [11,12,13,14,15,16,17,18,19,20,21,22,23,24,25,26,27] and add details on effects such as lattice vibrations [11,12,13], Fermi resonance [14,15,16,17,18,19] and Davydov splitting [20] in addition to the evidence of different crystalline forms [21,22,23]. However, to the best of our knowledge, there are no reports of the inelastic neutron scattering (INS) spectra of CH_2_Cl_2_ or CCl_4_ systems. In the case of CHCl_3_, its INS spectrum was reported in a study of hydrogen bonding in chloroform-acetone mixtures [28].

INS spectroscopy provides a unique assessment of the structural dynamics that is not amenable from its optical counterparts, infrared and Raman spectroscopies. Due to the absence of selection rules, all vibrational modes are potentially observable through INS spectroscopy. Large amplitude/low wavenumber modes, including lattice modes, usually problematic for optical spectroscopy, tend to yield intense bands in INS spectra. In addition, INS spectroscopy is particularly suitable for the computational spectroscopy approach. The INS intensity of a band associated with a given vibrational mode is proportional to the neutron scattering cross-section of the moving nuclei and to the amplitude of nuclei displacement. While the former is an invariant experimental value, the latter is accessible from standard vibrational frequency calculations. In particular, DFT calculations—either periodic or discrete—were found to be highly efficient in predicting the eigenvectors (atomic displacements) for the vibrational normal modes and, thus, to simulate the corresponding INS spectrum (see, e.g., the above-mentioned work from some of the authors [1,2,3,4,5,6,7,8]).

In this work, the INS spectra of the compounds CCl_4_, CHCl_3_ and CH_2_Cl_2_ in the solid state were collected at TOSCA—a time-of-flight INS spectrometer at the ISIS Pulsed Neutron and Muon Facility—and compared with the corresponding simulated spectra, obtained from periodic DFT calculations. TOSCA does not provide relevant information for the high wavenumber region—for which the infrared and Raman counterparts deliver better results—but has good resolution in the low wavenumber region, with good quality up to ca. 2000 cm^−1^. The excellent agreement between experimental and calculated spectra allows a confident assignment of the vibrational features, including, not only, the molecular fundamental modes but also lattice modes and combination modes.

## 2. Results and Discussion

### 2.1. Tetrachloromethane—CCl_4_

**Figure 1:**
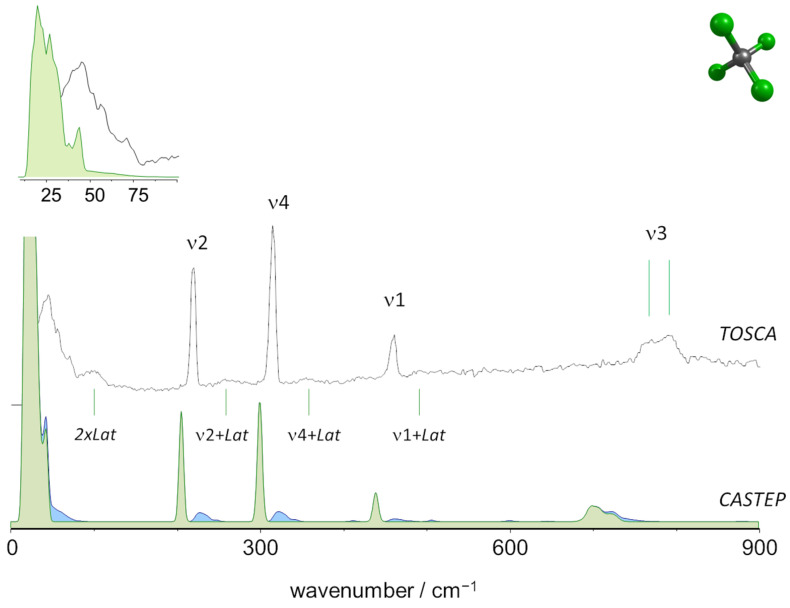
The INS spectra of tetrachloromethane (carbon tetrachloride) up to 900 cm^−1^: Experimental (top, TOSCA) and simulated from periodic calculations (bottom, CASTEP). Colors indicate the intensity contributions from fundamental modes (green) and two-quanta events (blue). “Lat” stands for librational and translational lattice modes. Inset: detail of the low wavenumber region.

Carbon tetrachloride is known to form three solid phases on cooling from the liquid at ambient pressure: a face-centered cubic phase Iα, a rhombohedral phase Iβ, and a monoclinic phase II. According to Rudman and Post [29], the monoclinic is the stable form below 225 K and, thus, should be considered to be present at the INS experiment conditions (ca. 10 K). As can be seen from Figure 1 the computational results provide an excellent description of the molecular modes. The same good qualitative agreement has already been reported by Tamarit et al. [30] with periodic-DFT calculations using a functional supplemented with a dispersion correction (PBE-G06). The low wavenumber region, shown in the inset, discloses the differences between both calculations: in the absence of dispersion correction (this work, shown in the inset), translational and libration modes span over the 11–46 cm^−1^ range, while Tamarit et al. [30] report “a large number of branches lying between ≈2 and ≈9 meV” (16–72 cm^−1^). This comparison allows the assignment of the most prominent features in the band profile, particularly the lattice mode at ca. 70 cm^−1^, which can be described as a translational mode (the molecular center of mass is displaced from its equilibrium position).

For the four intramolecular modes, both the positions and intensities (particularly the relative intensities) are correctly predicted, with a near-perfect match between calculated and observed values. The calculated combinations involving lattice modes (blue intensities in Figure 1) find correspondence with the low-intensity broad profiles observed in the experimental spectrum.

The doublet observed in the region of ν3 (INS band maxima at ca. 765 and 790 cm^−1^) deserves further discussion. A similar doublet is present in the infrared and Raman spectra and has been the subject of some attention [17,18,19]. It is generally ascribed to a Fermi resonance interaction between the fundamental ν3 and the combination ν1 + ν4 in the gas and in condensed phases, although this assignment for the crystalline state is not consensual. Tse and Lin [12] and Clark and Hunter [16] ascribe the presence of the doublet in the vibrational spectra of crystalline CCl_4_ to factor group splitting. Nevertheless, the comprehensive work of Chakraborty and Rai [19] supports the assignment of the doublet to ν3/(ν1 + ν4) Fermi resonance interaction, with the low wavenumber band being the “fundamental” and the high wavenumber the “ν1 + ν4” component. Tamarit et al. [30] note that periodic DFT calculations predict a broad band centered at about 89 meV (ca. 718 cm^−1^), which they ascribe to “the resolved multiplet in the experimental spectrum centered at some 96 meV“ (ca. 775 cm^−1^), without considering Fermi resonance effects. The periodic DFT calculations herein reported are more specific in predicting the splitting of the ν3 mode, giving rise to the doublet profile observed in Figure 1 due to the crystal splitting effect (of course, as the frequency calculations are performed within the harmonic approximation, anharmonic effects such as Fermi resonance are not predicted by the calculations). The analysis of INS intensities within the doublet is not compatible with the Fermi resonance effect, since the high wavenumber band—assumed to be the “intensity stealing” ν1 + ν4 combination component—has higher intensity than the band with more fundamental character, at lower wavenumber. The possible coincidence of effects, i.e., crystal splitting and Fermi resonance, although possible, is expected to give rise to a more complex multiplet. In this way, the herein reported results support the assignment of the 765–790 cm^−1^ doublet to crystal splitting (calculated atomic displacements for lower and higher wavenumber components of ν3 are shown in Figure S1c,d, respectively, Supplementary Materials).

### 2.2. Trichloromethane—CHCl_3_

**Figure 2:**
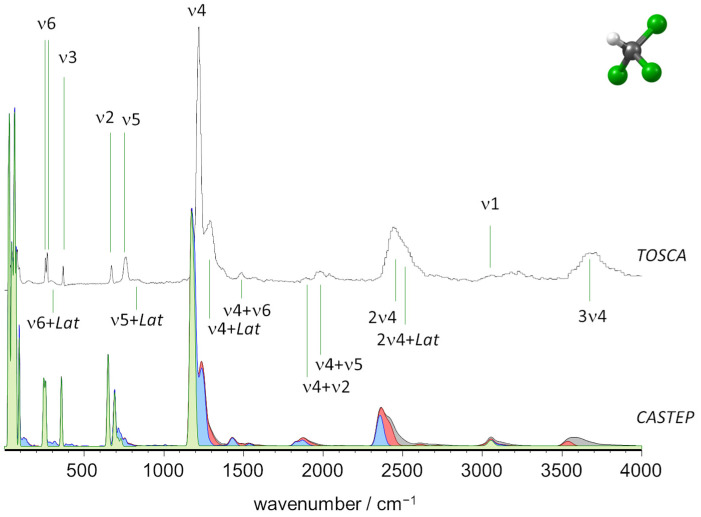
The INS spectra of trichloromethane (chloroform) up to 4000 cm^−1^: Experimental (top, TOSCA) and simulated from periodic calculations (bottom, CASTEP). Colors indicate the intensity contributions from fundamental modes (green), two-quanta events (blue), three-quanta events (red), and higher-order quantum events (grey).

Figure 2 compares the experimental and calculated INS spectra of CHCl_3_. There is a remarkable agreement between calculated and experimental spectra in both frequencies and intensities. For the fundamental modes, calculations correctly predicted the splitting of the ν6 mode (observed at ca. 257–268 cm^−1^) and the low intensity of ν1 (barely observed at ca. 3060 cm^−1^). More impressive was the remarkable prediction of the multi-quanta sequence based on ν4, extending to 3680 cm^−1^. In addition to the 1 × ν4, 2 × ν4 and 3 × ν4 sequence, there were also two-quanta combinations of ν4 with ν2, ν5 and ν6 (Figure 2).

In addition, the profile on the higher wavenumber side of the bands associated with ν4, 2 × ν4 and 3 × ν4 transitions was correctly described by the presence of multi-quanta combinations with lattice vibrations, i.e., with both translational and librational modes. 

**Figure 3:**
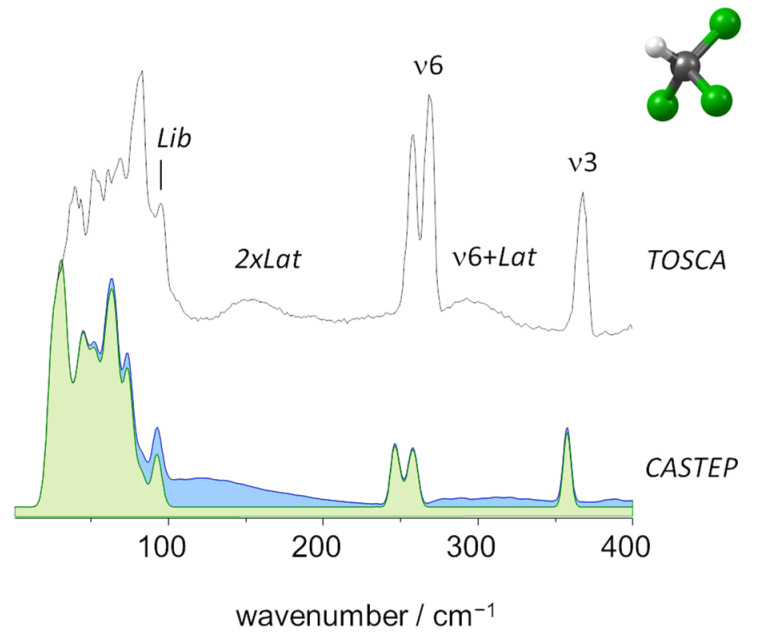
The INS spectra of trichloromethane (chloroform) in the low wavenumber region, up to 400 cm^−1^: Experimental (top, TOSCA) and simulated from periodic calculations (bottom, CASTEP). Colors indicate the intensity contributions from fundamental modes (green) and two-quanta events (blue).

In fact, while for CH_2_Cl_2,_ only librational motions appear with a significant contribution to the multi-quanta modes (as it will be discussed later), in the case of CHCl_3_, both librations and translations were found to contribute to the combination bands. This effect may be related to the stronger C-H···Cl contacts in CHCl_3_. The highest wavenumber lattice mode, at ca. 93 cm^−1^ and identified in Figure 3 below and Figure S2c, Supplementary Materials, due to its uniqueness in both experimental and calculated spectra, is illustrative. Although it is mostly librational in nature, it involves a significant stretching of the C-H···Cl distances between neighboring molecules in the crystal.

### 2.3. Dichloromethane—CH_2_Cl_2_

**Figure 4:**
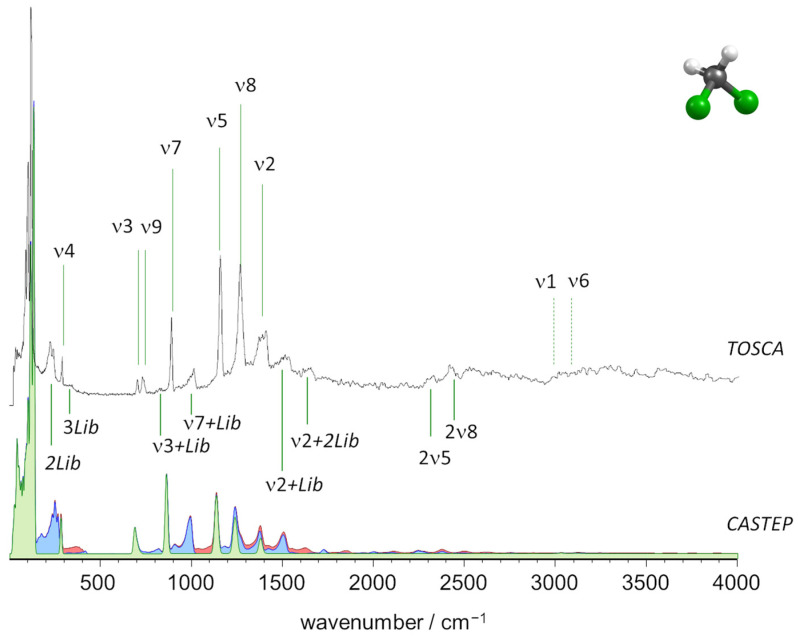
The INS spectra of dichloromethane up to 4000 cm^−1^: Experimental (top, TOSCA) and simulated from periodic calculations (bottom, CASTEP). Colors indicate the intensity contributions from fundamental modes (green), two-quanta events (blue) and three-quanta events (red).

Figure 4 compares the experimental and calculated INS spectra of CH_2_Cl_2_. In the calculated spectrum, the fundamental bands are shown in green, while the two-quanta and three-quanta contributions are shown in blue and red, respectively.

**Figure 5:**
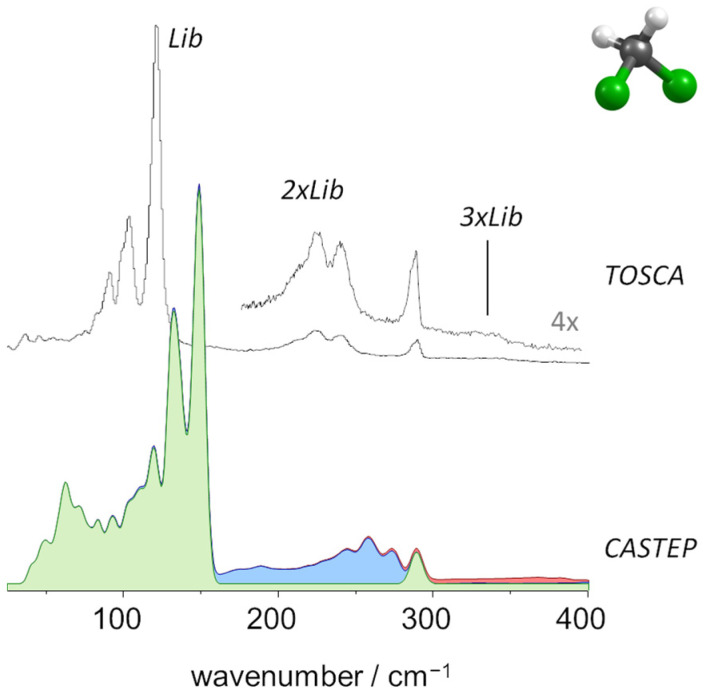
Detailed view of the INS spectra of dichloromethane in the low wavenumber region, up to 400 cm^−1^: Experimental (top, TOSCA) and simulated from periodic calculations (bottom, CASTEP). Colors indicate the intensity contributions from fundamental modes (green), two-quanta events (blue) and three-quanta events (red).

As mentioned above, TOSCA has limited capabilities for the high wavenumber region (above 2000 cm^−1^), and ν1 and ν6 modes (CH stretching) were not observed clearly. However, TOSCA’s resolution at the lower wavenumber end is excellent and disclosed several bands arising from large amplitude/low wavenumber vibrational modes. Figure 5 presents a closer look at the region below 400 cm^−1^, which includes the intermolecular (librational and translational) modes. 

The immediate evidence from Figure 4 and Figure 5 is the remarkable agreement between the experimental spectrum and the one simulated from periodic DFT calculations. The fundamental modes were easily identified at the expected wavenumbers. The presence of bands arising from multi-quanta transitions involving the librational modes is manifested in Figure 4. The twelve librational modes of the CH_2_Cl_2_ crystal (Z = 4) give rise to three sharp and intense bands at 92, 103 and 121 cm^−1^ (see Figure 5) and to their first and second overtones (2 × Lib and 3 × Lib) observed at ca. 250 cm^−1^ and ca. 380 cm^−1^, respectively. Multi-quanta transitions combining fundamental and librational modes were observed across the spectrum, but with particular relevance at ca. 1015 and 1515 cm^−1^, associated with ν7 (CH_2_ wag) and ν2 (CH_2_ scissor), respectively.

## 3. Experimental

Compounds: Dichloromethane (99%), trichloromethane (chloroform, 99%) and tetrachloromethane (carbon tetrachloride, 99%) were purchased from Sigma-Aldrich (Gillingham, Dorset, UK) and used as received.

INS spectroscopy: Inelastic neutron scattering experiments were performed with the TOSCA spectrometer, an indirect geometry time-of-flight spectrometer at the ISIS Neutron and Muon Source at theSTFC Rutherford Appleton Laboratory (Chilton, UK) [31,32,33,34]. The samples, with a total amount of ca. 2 g, were packed inside flat thin-walled aluminum cans of 5 cm height by 4 cm width, with a path length of 2 mm, which were mounted perpendicular to the beam using a regular TOSCA center stick. Spectra were collected below 15 K, measured for the 16 to 8000 cm^−1^ energy-transfer range. The resolution is ΔE/E ≈ 1.25%. Data were converted to the conventional scattering law, S(Q,ω) vs. energy transfer (in cm^−1^), using the MANTID program (version 4.0.0) [35].

Quantum chemical calculations: Periodic density functional theory (DFT) calculations were carried out using the plane wave pseudopotential method, as implemented in the CASTEP code (version 17.21) [36,37]. Exchange and correlation were approximated using the PBE [38] functional within the generalized gradient approximation (GGA). This method was found to provide a good compromise between accuracy and computational time for both frequency and intensity calculations in molecular crystals (see, e.g., discussion in [5,8]). The plane-wave cut-off energy was 830 eV. Brillouin zone sampling of electronic states was performed on 7 × 4 × 3 (16 k-points), 4 × 3 × 5 (12 k-points) and 4 × 4 × 2 (10 k-points) Monkhorst-Pack grids for CH_2_Cl_2_, CHCl_3_ and CCl_4_, respectively. The equilibrium structure, an essential prerequisite for lattice dynamics calculations, was obtained by BFGS geometry optimization, after which the residual forces converged to ±0.00087 eV Å^−1^.

The initial structures were taken from the reported CCDC crystal structures: DCLMET11 (CH_2_Cl_2_ [39]), CLFORM01 (CHCl_3_ [40]) and CARBTC07 (CCl_4_ [41]), and cell parameters were kept constant during geometry optimization. In the case of CCl_4_, calculations were performed for a crystal lattice structure adjusted by symmetry defaulting to a primitive unit cell with one-half of the 32 molecules in the original unit cell.

Phonon frequencies were obtained by diagonalization of the dynamical matrix, computed using density-functional perturbation theory [27], to compute the dielectric response and the Born effective charges, and, from these, the mode oscillator strength tensor and infrared absorptivity were calculated. The atomic displacements in each mode, which are part of the CASTEP output, enable visualization of the modes to aid assignments and are also all that is required to generate the INS spectrum using the program aCLIMAX (version 6.0.0 LE) [42]. It is emphasized that, for the calculated spectra shown, the transition energies have not been scaled. aCLIMAX allows the “deactivation” of selected vibrational modes, a procedure used to unambiguously assign the observed multi-quanta features (overtone and combination bands).

## 4. Conclusions

INS spectroscopy has a synergic combination with periodic DFT calculations, which, in the present case, have resulted in outstanding agreement between calculated and experimental INS spectra. This has allowed not only the confirmation of the fundamental molecular modes, but also the detailed description of overtone and combination bands. Taking advantage of the excellent resolution of the TOSCA instrument at the lower wavenumber region, several lattice modes were assessed. A controversial assignment of a doublet found in the INS spectrum of CCl_4_ was discussed in view of its description within the harmonic oscillator approximation.

## Data Availability

Not applicable.

## Supplementary Materials

The following supporting information can be downloaded at: https://www.mdpi.com/article/10.3390/molecules27217661/s1, Table S1. Calculated and experimental INS wavenumbers of tetrachloromethane, CCl_4_; Table S2. Calculated and experimental INS wavenumbers of trichloromethane, CHCl_3_; Table S3. Calculated and experimental INS wavenumbers of dichloromethane, CH_2_Cl_2_. Figure S1. Atomic displacements of some vibrational modes of carbon tetrachloride, CCl_4_; Figure S2. Atomic displacements of some vibrational modes of trichloromethane, CHCl_3_; Figure S3. Atomic displacements of some vibrational modes of dichloromethane, CH_2_Cl_2_.

## References

[1] Araujo C.F., Coutinho J.A.P., Nolasco M.M., Parker S.F., Ribeiro-Claro P.J.A., Rudić S., Soares B.I.G., Vaz P.D. (2017). Inelastic neutron scattering study of reline: Shedding light on the hydrogen bonding network of deep eutectic solvents. Phys. Chem. Chem. Phys..

[2] Vilela C., Freire C.S.R., Araújo C., Rudić S., Silvestre A.J.D., Vaz P.D., Ribeiro-Claro P.J.A., Nolasco M.M. (2020). Understanding the structure and dynamics of nanocellulose-based composites with neutral and ionic poly(methacrylate) derivatives using inelastic neutron scattering and DFT calculations. Molecules.

[3] Araujo C.F., Nolasco M.M., Ribeiro-Claro P.J.A., Rudić S., Silvestre A.J.D., Vaz P.D., Sousa A.F. (2018). Inside PEF: Chain Conformation and Dynamics in Crystalline and Amorphous Domains. Macromolecules.

[4] Nolasco M.M., Araujo C.F., Vaz P.D., Amado A.M., Ribeiro-Claro P. (2020). Vibrational dynamics of crystalline 4-phenylbenzaldehyde from INS spectra and periodic DFT calculations. Molecules.

[5] Ribeiro-Claro P.J.A., Vaz P.D., Nolasco M.M., Gil F.P.S.C., de Carvalho L.A.E., Marques M.P.M., Amado A.M. (2021). New Insights on the Vibrational Dynamics of 2-Methoxy-, 4-Methoxy- and 4-Ethoxy-Benzaldehyde from INS Spectra and Periodic DFT Calculations. Materials.

[6] Adilina I.B., Aulia F., Fitriady M.A., Oemry F., Widjaya R.R., Parker S.F. (2020). Computational and spectroscopic studies of carbon disulfide. Molecules.

[7] Zachariou A., Hawkins A.P., Collier P., Howe R.F., Lennon D., Parker S.F. (2020). The methyl torsion in unsaturated compounds. ACS Omega.

[8] Ribeiro-Claro P.J.A., Vaz P.D., Nolasco M.M., Amado A.M. (2018). Understanding the vibrational spectra of crystalline isoniazid: Raman, IR and INS spectroscopy and solid-state DFT study. Spectrochim. Acta Part A Mol. Biomol. Spectrosc..

[9] Barone V., Alessandrini S., Biczysko M., Cheeseman J.R., Clary D.C., McCoy A.B., DiRisio R.J., Neese F., Melosso M., Puzzarini C. (2021). Computational molecular spectroscopy. Nat. Rev. Methods Prim..

[10] Shimanouchi T. (1977). Tables of Molecular Vibrational Frequencies. Consolidated Volume II. J. Phys. Chem. Ref. Data.

[11] Anderson A., Andrews B., Torrie B.H. (1985). Raman and infrared studies of the lattice vibrations of some halogen derivatives of methane. J. Chim. Phys..

[12] Anderson A., Torrie B.H., Tse W.S. (1979). Raman and far infrared spectra of the solid phases of carbon tetrachloride. Chem. Phys. Lett..

[13] Anderson A., Torrie B.H., Danagher D.J., Laurin D.G., White J.K., Zung W.W.E. (1986). Raman and far-infrared spectra of crystalline methylene chloride. J. Raman Spectrosc..

[14] Shurvell H.F. (1971). The Raman spectrum of solid CCl4 and isotopically enriched C35Cl4. Spectrochim. Acta Part A Mol. Spectrosc..

[15] Cook C.F., Person W.B., Hall L.C. (1967). Absolute infrared intensities of the fundamental absorption bands in solid CCl4. Spectrochim. Acta Part A Mol. Spectrosc..

[16] Clark R.J.H., Hunter B.K. (1971). Raman spectra and factor-group analyses of crystalline group IV tetrachlorides. J. Chem. Soc. A Inorg. Phys. Theor. Chem..

[17] Kenney J.T., Powell F.X. (1967). Difference bands in the raman spectrum of carbon tetrachloride. J. Chem. Phys..

[18] Abramowitz S., Comeford J.J. (1965). Fermi resonance in condensed CF4 and CCl4. Spectrochim. Acta.

[19] Chakraborty T., Rai S.N. (2006). Comparative study of infrared and Raman spectra of CCl4 in vapour and condensed phases: Effect of LO-TO splitting resulting from hetero-isotopic TD-TD interactions. Spectrochim. Acta Part A Mol. Biomol. Spectrosc..

[20] D’Alessio E.A., Dodero E., Pomposiello C. (1972). Infrared spectra in polarized light of crystalline chloroform. J. Chem. Phys..

[21] Dumas J.P. (1977). Evidence for 4 Crystalline Phases for Ccl4 at Atmospheric-Pressure. Comptes Rendus Hebd. Des Seances L Acad. Des. Sci. Ser. C.

[22] Dows D.A. (1961). Intermolecular coupling of vibrations in molecular crystals. II. Intermolecular forces in CH3Cl and CD3Cl. J. Chem. Phys..

[23] Brown C.W., Obremski R.J., Allkins J.R., Lippincott E.R. (1969). Vibrational spectra of single crystals and polycrystalline films of CH2Cl2 and CH2Br2. J. Chem. Phys..

[24] Andrews B., Anderson A., Torrie B. (1984). Raman and infrared spectra of crystalline chloroform. Chem. Phys. Lett..

[25] Jemmis E.D., Giju K.T., Sundararajan K., Sankaran K., Vidya V., Viswanathan K.S., Leszczynski J. (1999). An ab initio and matrix isolation infrared study of the 1:1 C2H_2_- CHCl_3_ adduct. J. Mol. Struct..

[26] Kimoto A., Yamada H. (1968). Infrared Spectra of Crystalline CHCl_3_ and CDCl_3_. Bull. Chem. Soc. Jpn..

[27] Tsyashch Y.P., Bankova L.E. (1965). Infrared Absorption of CHCl_3_-CHBr_3_ Mixed Crystals. Opt. Spectrosc..

[28] Vaz P.D., Nolasco M.M., Gil F.P.S.C., Ribeiro-Claro P.J.A., Tomkinson J. (2010). Hydrogen-bond dynamics of C-H···O interactions: The chloroform···acetone case. Chem. A Eur. J..

[29] Rudman R., Post B. (1966). Carbon tetrachloride: A new crystalline modification. Science.

[30] Moratalla M., Gebbia J.F., Ramos M.A., Pardo L.C., Mukhopadhyay S., Rudić S., Fernandez-Alonso F., Bermejo F.J., Tamarit J.L. (2019). Emergence of glassy features in halomethane crystals. Phys. Rev. B.

[31] Parker S.F., Lennon D., Albers P.W. (2011). Vibrational Spectroscopy with Neutrons: A Review of New Directions. Appl. Spectrosc..

[32] ISIS Facility INS/TOSCA. https://www.isis.stfc.ac.uk/Pages/tosca.aspx.

[33] Parker S.F., Fernandez-Alonso F., Ramirez-Cuesta A.J., Tomkinson J., Rudic S., Pinna R.S., Gorini G., Fernandez Castanon J. (2014). Recent and future developments on TOSCA at ISIS. Journal of Physics: Conference Series.

[34] Pinna R.S., Rudić S., Parker S.F., Armstrong J., Zanetti M., Škoro G., Waller S.P., Zacek D., Smith C.A., Capstick M.J. (2018). The neutron guide upgrade of the TOSCA spectrometer. Nucl. Instrum. Methods Phys. Res. Sect. A Accel. Spectrometers Detect. Assoc. Equip..

[35] Arnold O., Bilheux J.C., Borreguero J.M., Buts A., Campbell S.I., Chapon L., Doucet M., Draper N., Leal R.F., Gigg M.A. (2014). Mantid-Data analysis and visualization package for neutron scattering and mu SR experiments. Nucl. Instrum. Methods Phys. Res. A.

[36] Clark S.J., Segall M.D., Pickard C.J., Hasnip P.J., Probert M.J., Refson K., Payne M.C. (2005). First principles methods using CASTEP. Z. Fur Krist..

[37] Refson K., Tulip P.R., Clark S.J. (2006). Variational density-functional perturbation theory for dielectrics and lattice dynamics. Phys. Rev. B.

[38] Perdew J.P., Burke K., Ernzerhof M. (1996). Generalized gradient approximation made simple. Phys. Rev. Lett..

[39] Podsiadło M., Dziubek K., Katrusiak A. (2005). In situ high-pressure crystallization and compression of halogen contacts in dichloromethane. Acta Crystallogr. Sect. B Struct. Sci..

[40] Dziubek K., Podsiadlo M., Katrusiak A. (2009). Molecular Symmetry and Isostructural Relations in Crystal Phases of Trihalomethanes CHCl_3_, CHBrCl_2_, CHBr_2_Cl, and CHBr_3_. J. Phys. Chem. B.

[41] Cohen S., Powers R., Rudman R. (1979). Polymorphism of the crystalline methylchloromethane compounds. VI. The crystal and molecular structure of ordered carbon tetrachloride. Acta Crystallogr. Sect. B Struct. Crystallogr. Cryst. Chem..

[42] Ramirez-Cuesta A.J. (2004). aCLIMAX 4.0.1, The new version of the software for analyzing and interpreting INS spectra. Comput. Phys. Commun..

