# Supplementary material for: Structural Dynamics of Chloromethanes through Computational Spectroscopy: Combining INS and DFT
Source: Molecules. 2022 Nov 7;27(21):7661. doi: 10.3390/molecules27217661 (PMC9655087; doi:10.3390/molecules27217661)
Supplement: Supplementary file 1 [file molecules-27-07661-s001.zip › molecules-1998216-supplementary-2-of-2.pdf]

Structural Dynamics of Chloromethanes through Computational Spectroscopy: Combining INS and DFT

Mariela M. Nolasco[1*], Mariana Matos Coimbra[1], Stewart F. Parker[2], Pedro D. Vaz[3], and Paulo J. A. Ribeiro-Claro[1]

[1] CICECO—Instituto de Materiais de Aveiro, Departamento de Química, Universidade de Aveiro, 3810-193 Aveiro, Portugal
[2] ISIS Neutron & Muon Source, STFC Rutherford Appleton Laboratory, Chilton, Didcot, Oxfordshire OX11 0QX, UK
[3] Champalimaud Foundation, Champalimaud Centre for the Unknown, 1400-038 Lisboa, Portugal

Supplementary Material Document 2 of 2

Contents

Figure S1. – Atomic displacements of some vibrational modes of carbon tetrachloride, CCl_4.
Figure S2. – Atomic displacements of some vibrational modes of trichloromethane, $CHCl_3$.
Figure S3. – Atomic displacements of some vibrational modes of dichloromethane, CH_2Cl_2.

Figure S1. – Atomic displacements of some vibrational modes of carbon tetrachloride, CCl_4.

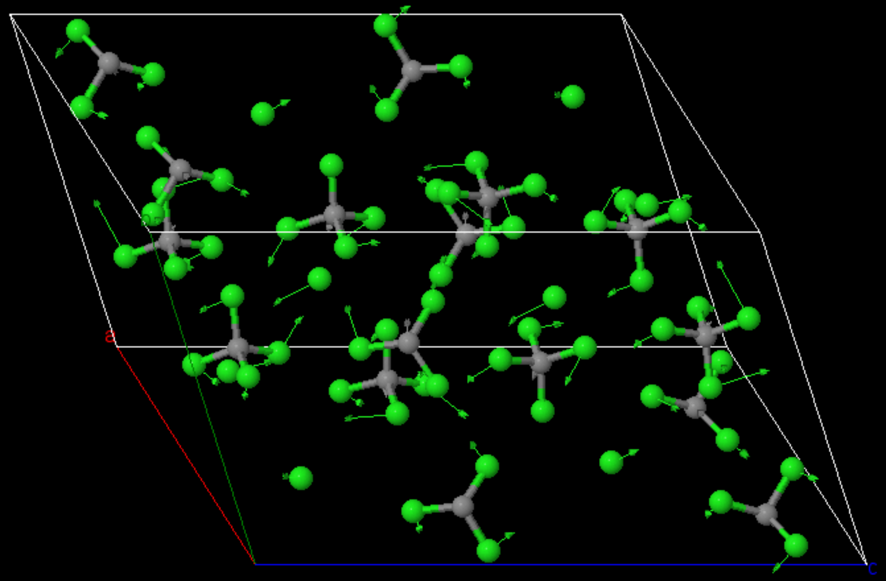

a) Librational mode, 20 cm^{-1}

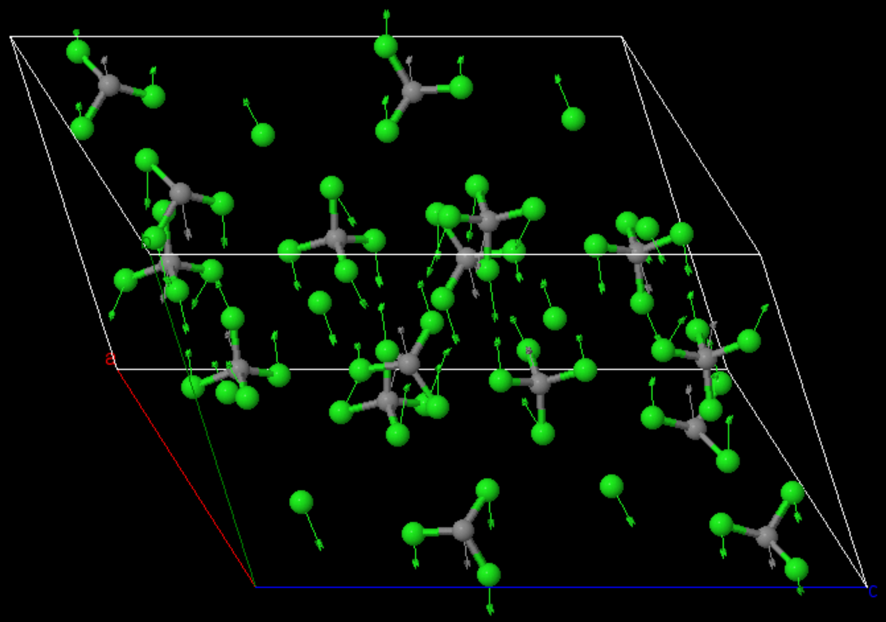

b) translational mode, 44 cm^{-1}

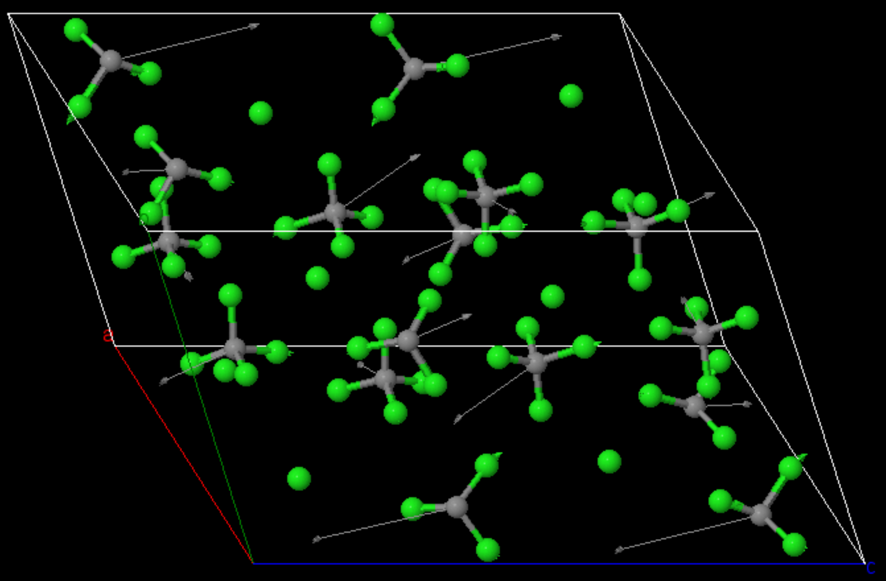

c) ν3 Lowest component

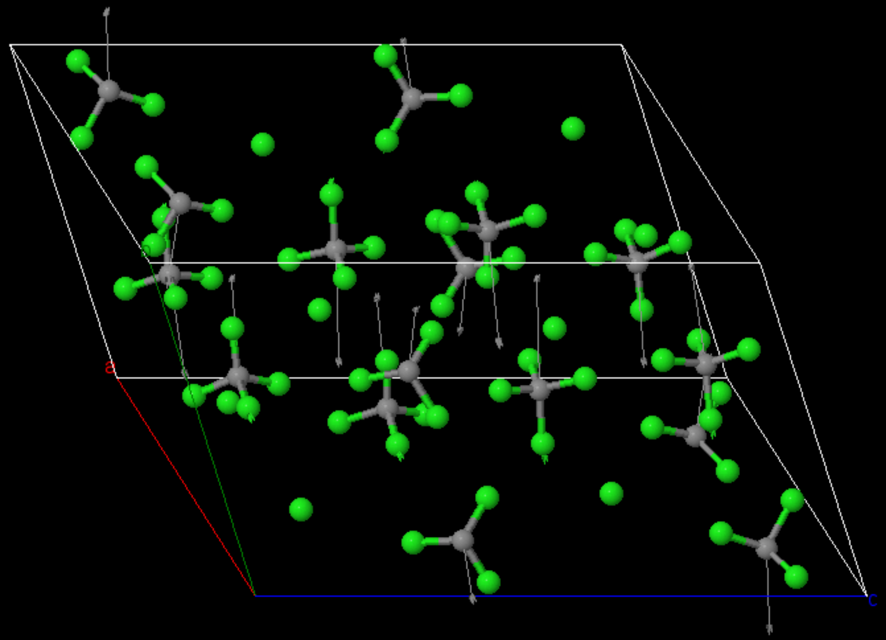

d) ν3 highest component

Figure S2. – Atomic displacements of some vibrational modes of trichlorometane, $CHCl_3$.

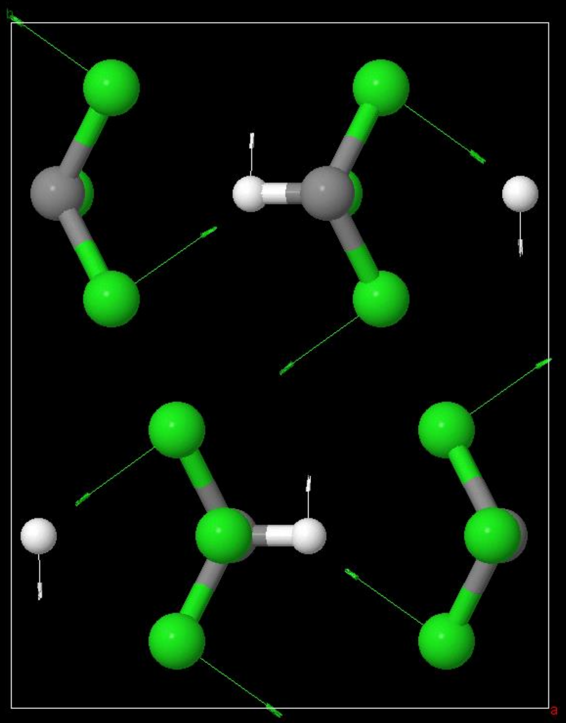

a) Librational mode, 45 cm^{-1}

Jmol

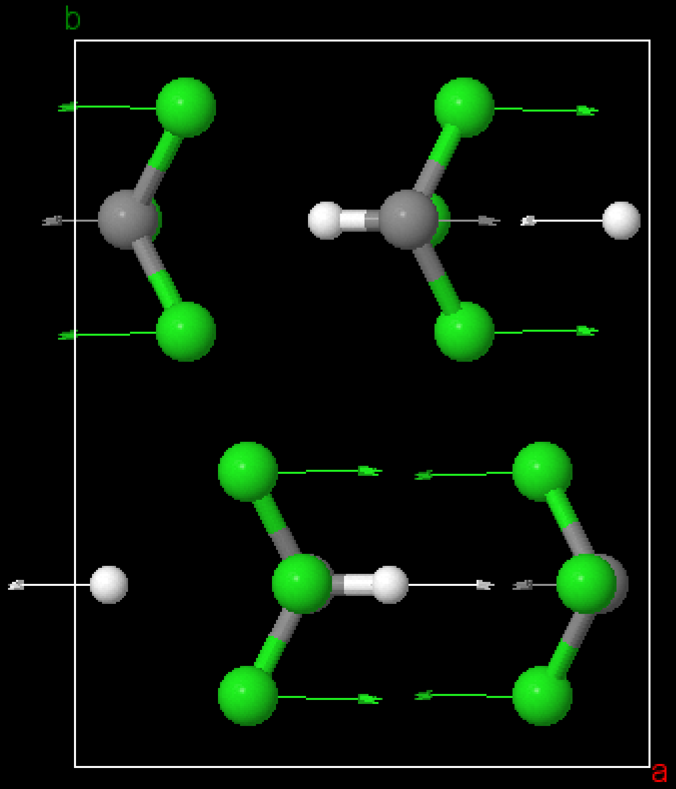

b) Translational mode, 66 cm^{-1}

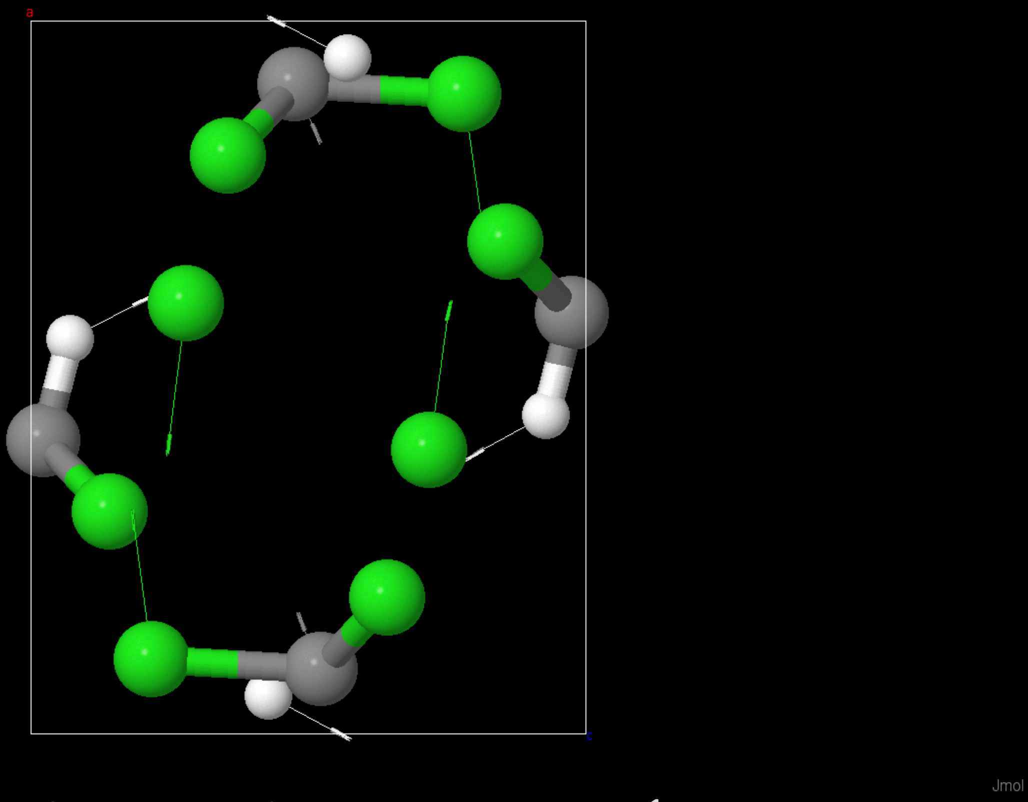

c) Librational mode, 93 cm^{-1}

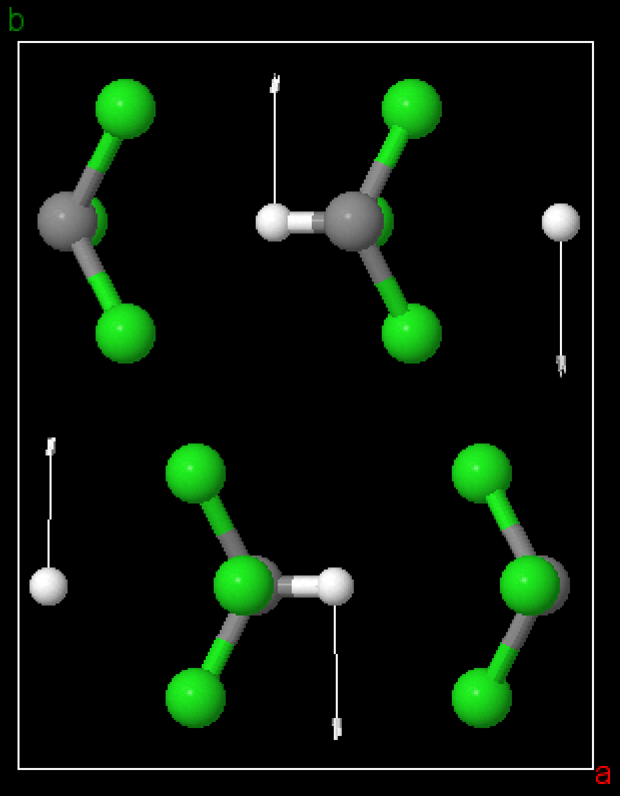

d) CH bending mode, 1180 cm^{-1}

Figure S3. – Atomic displacements of some vibrational modes of dichlorometane, CH_2Cl_2.

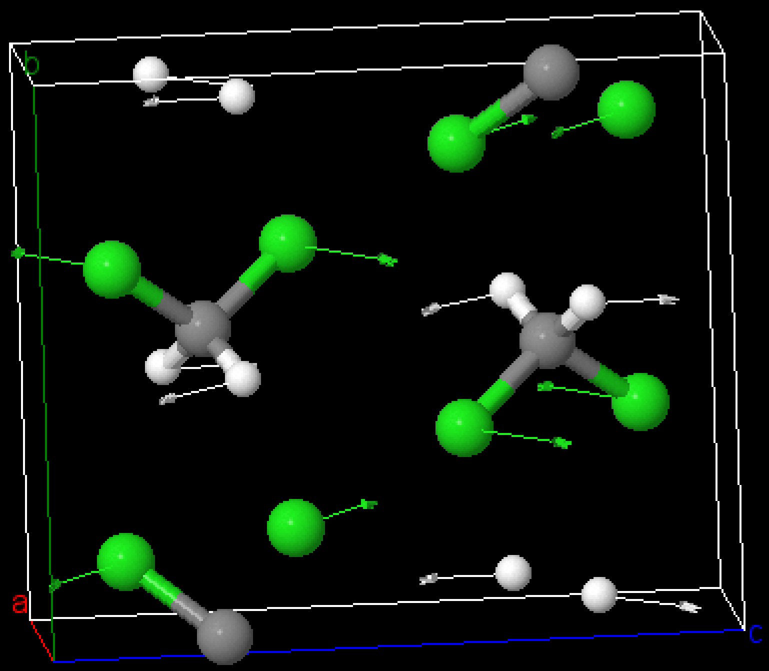

a) Librational mode, 117 cm^{-1}

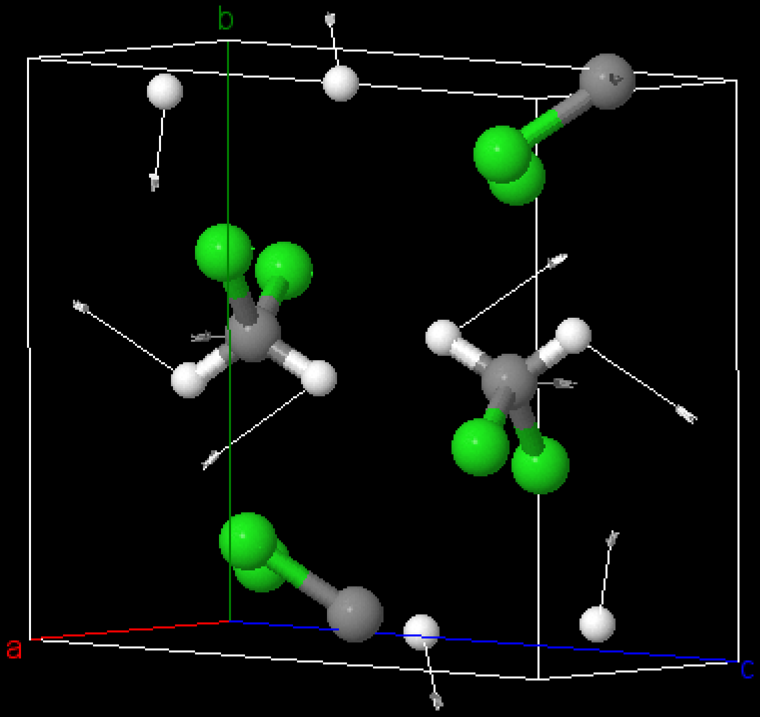

b) Librational mode, 132cm^{-1}

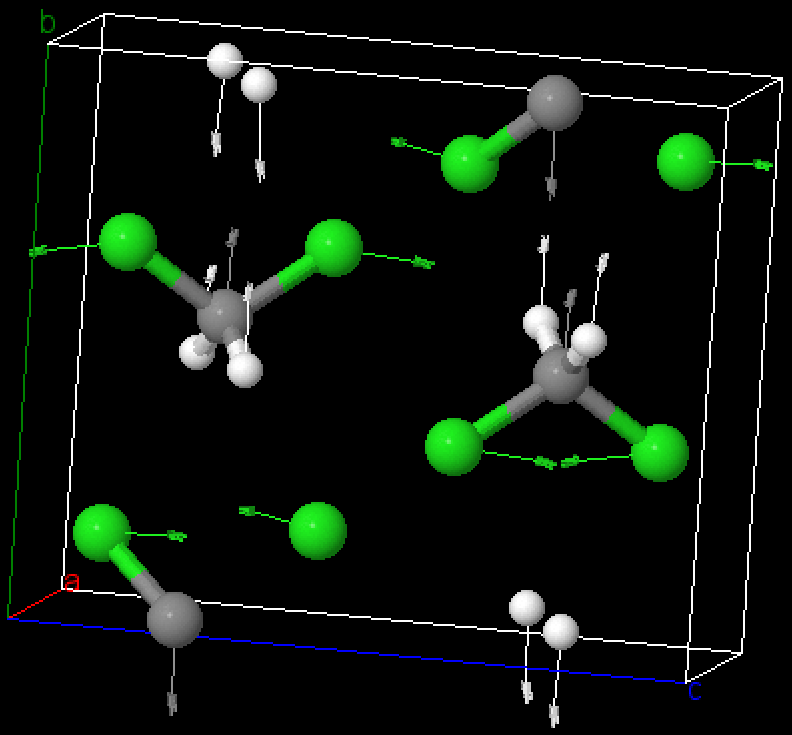

c) CCl_2 bending mode, 283 cm^{-1}

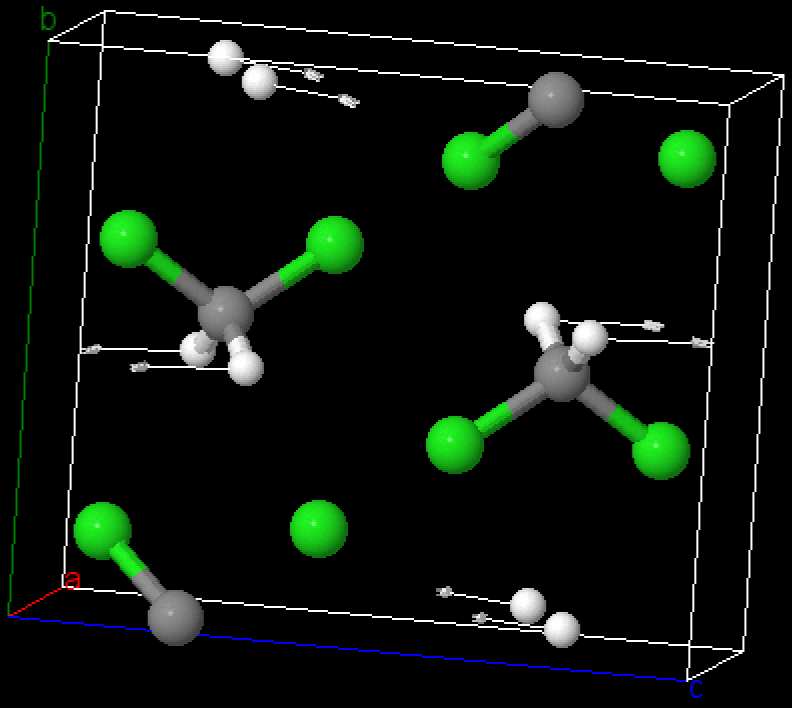

d) CH_2 rocking mode, 1230 cm^{-1}
