# Supplementary material for: Structural Dynamics of Chloromethanes through Computational Spectroscopy: Combining INS and DFT
Source: Molecules. 2022 Nov 7;27(21):7661. doi: 10.3390/molecules27217661 (PMC9655087; doi:10.3390/molecules27217661)
Supplement: Supplementary file 1 [file molecules-27-07661-s001.zip › molecules-1998216-supplementary-1-of-2.pdf]

Structural Dynamics of Chloromethanes through Computational Spectroscopy: Combining INS and DFT

Mariela M. Nolasco[1*], Mariana Matos Coimbra[1], Stewart F. Parker[2], Pedro D. Vaz[3], and Paulo J. A. Ribeiro-Claro[1]

[1] CICECO—Instituto de Materiais de Aveiro, Departamento de Química, Universidade de Aveiro, 3810-193 Aveiro, Portugal
[2] ISIS Neutron & Muon Source, STFC Rutherford Appleton Laboratory, Chilton, Didcot, Oxfordshire OX11 0QX, UK
[3] Champalimaud Foundation, Champalimaud Centre for the Unknown, 1400-038 Lisboa, Portugal

Supplementary Material

Document 1 of 2

Contents

Table S1. – Calculated and experimental INS wavenumbers of tetrachloromethane, CCl_4.

Table S2. – Calculated and experimental INS wavenumbers of trichloromethane, $CHCl_3$.

Table S3. – Calculated and experimental INS wavenumbers of dichloromethane, CH_2Cl_2.

Table S1. Calculated and experimental INS wavenumbers of tetrachloromethane, CCl_4.

CASTEP [1]	Experimental	Assignment	Approximate description
725/702	790/765	**ν3**	νCCl asym
440	460	**ν1**	νCCl sym
300	315	**ν4**	βCCl
206	219	**ν2**	βCCl
44	70	Lat	
20	45	Lat	

[1] Maxima in the INS simulated spectrum. ν and β stand for stretching and bending modes, respectively; Lat indicates a lattice mode, both translational or with translational and librational components.

Table S2. Calculated and experimental INS wavenumbers of trichloromethane, $CHCl_3$.

CASTEP [1]	Experimental	Assignment	Approximate description
3576	3680	3ν4	
3056	3060	**ν1**	νCH
2364	2444	2ν4	
1874	1975	ν4+ν5	
1824	1894	ν4+ν2	
1427	1484	ν4+ν6	
1237	1291	ν4+Lat	
1175	1218	**ν4**	βCH
691	756	**ν5**	νCCl_3 asym
650	670	**ν2**	νCCl_3 sym
357	368	**ν3**	βCCl_3 sym
257/247	268/258	**ν6**	βCCl_3 asym
93	94	Lat	
63	81	Lat	
45	50	Lib	

[1] Maxima in the INS simulated spectrum. ν and β stand for stretching and bending modes, respectively; Lib stands for pure librational mode, while Lat indicates a lattice mode, both translational or with translational and librational components.

Table S3. Calculated and experimental INS wavenumbers of dichloromethane, CH_2Cl_2.

CASTEP [1]	Experimental	Assignment	Approximate description
3117	n.o.	**ν6**	νCH_2 asym
3027	n.o.	**ν1**	νCH_2 sym
2373	2425	2ν8	
2240	2323	2ν5	
1623	1645	ν2+2Lib	
1504	1515	ν2+Lib	
1375	1390	**ν2**	βCH_2 scissor
1237	1270	**ν8**	βCH_2 rock
1135	1160	**ν5**	βCH_2 twist
994	1015	ν7+Lib	
862	892	**ν7**	βCH_2 wag
702	733	**ν9**	νCCl_2 asym
690	704	**ν3**	νCCl_2 asym
380	343	3Lib	
282	290	**ν4**	βCCl_2
265	240	2Lib	
250	224	2Lib	
133	121	Lib	
115	103	Lib	
101	92	Lib	

[1] Maxima in the INS simulated spectrum. ν and β stand for stretching and bending modes, respectively; Lib stands for pure librational mode.
